# Functional and Nutritional Properties of Proteins From Barley Malting By‐Products

**DOI:** 10.1111/1750-3841.70624

**Published:** 2025-10-28

**Authors:** Niccolò Pilla, Elisa De Arcangelis, Gianfranco Pannella, Emanuele Marconi

**Affiliations:** ^1^ Dpt. Science and Technology for Sustainable Development and One Health Università Campus Bio‐Medico di Roma Rome Italy; ^2^ Dpt. of Agricultural, Forest and Food Sciences – DISAFA Università degli Studi di Torino Grugliasco Italy; ^3^ Council for Agricultural Research and Economics (CREA) Research Centre for Food and Nutrition Rome Italy

**Keywords:** alternative proteins, barley, by‐product, malt rootlets, techno‐functional

## Abstract

Nowadays, global demand for alternative protein sources is rising, and exploitation of by‐products can concurrently meet the challenges of integrating sustainability and circular economy in the food industry. Barley malt rootlets represent the main by‐product of the malting industry and are normally discarded as animal feed. In this work, the protein concentrates obtained after alkali extraction of industrial by‐products were characterized (amino acid composition, SDS page and protein digestibility) with particular regard to the techno‐functional properties (solubility, foaming and emulsifying abilities, water holding capacity (WHC), and fat absorption capacity). Data were further compared with those obtained on common plant‐derived proteins such as soy, pea, and rice, to elucidate the potential application of proteins from malt rootlets in novel food formulations. Nutritionally, significant levels of lysine were detected (–7.0 g/100 g proteins) with comparable digestibility data to other legume protein sources (–75%). The solubility trend aligned with soy and pea proteins, being close to 90% in the pH range 11–12. Foaming and emulsifying abilities were weaker than soy and pea proteins, while WHC (–6 mL/g) exceeded that of other proteins thus evidencing its potential in liquid or semi‐liquid formulations. Overall, both malt rootlets and malt rootlets pellet, the true industrial by‐product, can potentially be up‐cycled materials for protein rich ingredients with suitable nutritional and technological performance.

**Practical Applications:**

The study provides an insight into the functional properties of proteins in barley malt rootlets.

Results support the up‐cycle of brewing by‐products for the recovery of high‐proteins ingredients

## 1 Introduction

By 2050, the world population is projected to reach 9.1 billion, driving an urgent need to increase food production by approximately 70% (FAO 2009) to ensure global food security. In this context, the worldwide rise in animal protein demand is expected to lead to significant environmental consequences, due to its meaningful contribution to global greenhouse gas emissions (Gil et al. 2024; Henchion et al. 2017), thus incremental substitution of animal‐derived proteins with plant‐based alternatives could substantially help mitigate this scenario (Mazac et al. 2022; McGregor et al. 2024). The growing interest in plant‐based diets, supported by the greater sensitivity towards animal welfare and sustainability aspects, laid the foundation for a sharp market increase in alternative proteins, which is expected to reach 26.5 billion USD by 2030 (Malila et al. 2024). At the same time, to meet growing global food demand, the agrifood supply chain will need to expand its productivity, resulting in increased food waste. Nowadays, it is estimated that nearly one third of food produced for human consumption is discarded, amounting to roughly one billion tons annually (Dhiman et al. 2025). In this context, the circular economy provides a theoretical framework aimed at encouraging, supporting, and developing strategies to curb excessive raw material consumption and minimize waste through regeneration, reuse, and innovation (Maqbool et al. 2020). Indeed, food residues hold potential as sources of bioactive compounds, and their recovery can help meet sustainability goals and create new bioactive ingredients (Olivares‐Galván et al. 2022a). Barley is the fourth most cultivated cereal and the major grain used in brewing (Mahalingam 2018), beyond being conventionally utilized for animal fodder (–66%) or for food applications (–2%) (Baik and Ullrich 2008). It has also gained interest due to its nutritional advantages regarding the beta‐glucan content (De Arcangelis et al. 2019a; De Arcangelis et al. 2019b; Cuomo et al. 2022; Jaeger et al. 2023; Lyu et al. 2022).

Brewing by‐products possess significant nutritional value and may serve as cost‐effective, accessible raw materials for various applications, including nutraceuticals and functional foods (Karlović et al. 2020). Malting is the initial step in the brewing process, consisting of a controlled germination, where several enzymes are produced or activated, causing the breakdown of significant biopolymers like proteins, starch, and cell‐wall polysaccharides. Barley malt rootlets are formed during the germination stage in the malting process, and are removed with the deculming operation after the kilning step, since they confer a bitter and astringent flavor to beer. Malt rootlets are normally referred to as the material removed after malt drying (Karlović et al. 2020; Neylon et al. 2020) nonetheless, in malting plants, malt rootlets include both the product from malting cereals germination and malt cleaning, consisting of rootlets, cereal fines, husks and small broken malted cereal grains (Commission Regulation (EU) 2017/1017). Because of their low bulk density, this material is further converted into pellets to reduce volume and preserve its quality during storage.

It is estimated that for every 100 kg of malt, 3–5 kilograms of malt rootlets are produced and transported to the feeding industry. The chemical composition of malting rootlets depends on the barley genotypes and mostly on the germination conditions (Donkor et al. 2012). Moisture content is low (4.2–12.9%), dietary fiber is mainly insoluble and includes arabinoxylans, moreover rootlets are a source of other different bioactive compounds (Karlović et al. 2020; Neylon et al. 2020), such as phenolic compounds (Budaraju et al. 2018).

In terms of protein content, malt rootlets reach approximately 20–40% on dry matter, while providing considerable amount of the essential amino acids isoleucine, phenylalanine, lysine, and leucine (Neylon et al. 2020; Robbins and Pomeranz 1971; Salama et al. 1997).

The study of protein functionalities (e.g. solubility, fat and water binding capacity, foaming capacity (FC), and stability) and nutritional properties is of paramount importance to incorporate a new protein source into food products or substite it in an existing recipe, so it is essential to assess its functionalities against those of reference proteins (Hewage et al. 2022).

Various extraction methods can be employed to isolate proteins. Among these, alkaline‐isoelectric precipitation is the most rapid and simple procedure for protein extraction from plant‐based material, that found wide scalability at the industrial level (Hadidi et al. 2023). It consists of solubilization of the material in alkaline solutions (pH 8–12) and adjustment to acidic conditions to favor protein aggregation (Hadidi et al. 2023). Differently from other brewing by‐products, such as brewers’ spent grains (Jaeger et al. 2023; Naibaho et al. 2022), proteins from barley malt rootlets have been less characterized, mainly addressing the optimization of parameters using different techniques and extractive liquids (Cheng et al. 2016; Olivares‐Galván et al. 2022b; Hernández‐Corroto et al. 2023).

The aim of this study was to characterize the protein fraction of both by‐products obtainable at the industrial level, Balrey malt rootlets (MR), produced after malt deculming step, and barley malt residual pellet (MRP) (which includes rootlets, husks, and broken malt grains). In particular, the techno‐functional properties of concentrated proteins after alkali‐isoelectrical precipitation were assessed in comparison with the three most common (Phillips 2017) commercial protein concentrates soy, pea and rice, to outline possible applications in the food industry.

## 2 Material and Methods

### 2.1 Samples

Barley malt rootlets (MR), obtained after the malt deculming step, and barley malt residual pellet (MRP), were kindly provided by S.a.p.l.o S.p.A (Pomezia, Italy), while three commercial protein powders, soy bean, pea and rice, (average protein content: 90% for soy and pea, and 80% for rice) were selected for the comparison of nutritional and functional properties.

### 2.2 Alkaline Extraction and Protein Quantification

Proteins were extracted according to alkaline extraction followed by isoelectric precipitation (Cheng et al. 2016; Gerliani et al. 2019; Du et al. 2018). Barley MR and MRP were finely ground, then about 10 g of sample powder was dispersed in 100 mL of Milli‐Q water. The pH was adjusted with 1 M NaOH to a final value of 11. Alkaline extraction was performed for 1 h at 40°C with continuous stirring. The suspension was then collected and centrifuged at 8000 rpm, 30 min, 4°C. The supernatant was collected, and the pH was adjusted to 3 with 1 M HCl for the protein precipitation. After 1 h of decanting the suspension was centrifuged (8000 rpm, 30 min, 4°C) and the supernatant discarded. The precipitate was washed with Milli‐Q water two times. The protein‐rich material was lyophilized and collected at 4°C for further analysis. The total amount of protein was calculated on malt rootlet by‐products and the related concentrated proteins (MR, MRP), using the Kjeldahl method with a nitrogen to protein conversion factor of 6.25 (Jaeger et al. 2023).

### 2.3 Amino Acid Profile

To determine the composition of amino acids a prior 24 h hydrolysis was performed using 6 N HCl (1 mL/mg protein) under anaerobic condition and at a temperature of 110°C (Gehrke et al. 1985). Aliquots were then dried, resuspended in 0.1 N HCl, and analyzed through HPAEC‐PAD (Messia et al. 2021). For chromatographic analysis, the samples were diluted and filtered (0.22 µm).The separation was performed with an AminoPac PA‐10 column equipped with a guard column, using eluents A = H_2_O, B = NaOH 250 mM, C = C_2_H_3_NaO_2_ 1 M, run time 48 min (0–12 min 85% A, 15% B; 12–16 min 80% A, 20%B; 16–24 min 68% A, 32% B; 24–36 min 36%A, 24%B, 40%C, 36–46 min 50%B, 50% C; 46–48 min 15%A, 85%B) with 30 min of conditioning at 85%A, 15%B. The flow rate was set at 0.25 mL/min at a temperature of 30°C. Detection was conducted with an electrochemical detector with a pH‐Ag/AgCl reference electrode and Au working electrode. The amino acids content was calculated using a calibration curve built with an amino acids standard solution (Sigma‐Aldrich AAS18).

### 2.4 SDS‐Page

Proteins of malt rootlets, and pea, soy, and rice isolates were analyzed by SDS‐PAGE under reducing conditions following the procedure of Fling and Gregerson (1986). Runs were carried out with precast gel 10% (BIO‐RAD, 4561034) and run with mini‐PROTEAN Tetra vertical electrophoresis unit in conjunction with PowerPac^TM^ basic Power supply (BIO‐RAD Laboratories). Proteins isolate were solubilized in a Tris‐HCl 100 mM pH 9.5, 1% SDS solution and then diluted with sample buffer (50 mM Tris–HCl, pH 6.8, 2 % (w/vol) SDS, 10 % (vol/vol) glycerol, 4.3 % (vol/vol) β‐mercaptoethanol, 0.0025 % (w/vol) bromophenol blue). Prior to electrophoresis, samples were heated at 100°C for 5 min and cooled to room temperature and 25 µL (∼30 µg protein) of each sample was loaded per well. The gels were run at 15 mA per gel for 70 min to completion. Gels were fixed and stained with Coomassie Blue G‐250 (BIO‐RAD, Bio‐Safe^TM^ 161–0876) for 45 min and destained with water. The gels were scanned and analyzed using Chemdoc software (BIO‐RAD, USA). Molecular weight markers included protein standard mixture ranging from 10 to 203 kDa (Sigma Aldrich MPSTD4). SDS‐PAGE analysis was done in triplicate.

### 2.5 Protein Digestibility

In vitro protein digestion (Tinus et al. 2012) was performed using a multienzyme solution consisting of about 16 mg trypsin (T4799 trypsin from porcine pancreas, lyophilized powder 1000–2000 BAEE units/mg solid), 31 mg chymotrypsin (C4129 chymotrypsin from bovine pancreas, Type II, lyophilized powder, 40 units/mg protein) and 13 mg of protease (P5147, protease from streptomyces griseus, Type XIV, 13 units/mg protein). The multi‐enzyme solution was prepared fresh on the day of analysis and kept at 37°C. An equivalent of 62.5 mg of protein was dispersed in 10 mL of Milli‐Q water and rehydrated at 37°C for 1 h, after that pH was adjusted to 8 with NaOH and/or 0.01 M HCl. Upon rehydration, 1 mL of the multienzyme solution was added to the 10 mL sample dispersion, and the pH of the digesta was recorded at time 0 and after 10 min. The pH decrease is caused by the free amino acid carboxyl groups from the protein chain released by the proteolytic enzymes during digestion. The change in pH at 10 min of digestion (ΔpH_10 min_) was used to calculate percent in vitro protein digestibility (IVPD) of the samples.

$$\mathrm{IVPD}=65.66+18.01\Delta pH_{10\;min}$$

### 2.6 Solubility

Relative protein solubility (PS) was determined at various pH values between 2 and 12, according to the protocol adapted from methods of Boye et al. (2010). In summary, 100 mg of protein isolate was dispersed in 10 mL of water. The pH was adjusted with 1 M NaOH and 1 M HCl. The suspension was left in continuous stirring for 1 h. After 1 h, the pH was corrected if needed, and then stirred for another 30 min. The suspensions were centrifuged (30 min at 2700 g), and the supernatants were collected. The amount of protein in the supernatant was determined by the method of Bradford (1976).

### 2.7 Foaming Properties

Foaming properties were expressed as foaming capacity (FC)Water and foam stability (FS) as described by Barac et al. (2015) with some modifications. An aliquot (30 mL) of protein solution 0.1% in water was placed in a graduated cylinder and foam was attained by whipping the solution for 1 min. Total foam volume was taken at 0 min for both parameters and at 3 min for FS.

Foaming properties were expressed as:

$$\begin{array}{ccc} \mathrm{FC}\left(\%\right) & = & \left(\text{volume after whipping}-\;\text{volume before whipping}\right)\\ & & /\left(\text{volume before whipping}\right)\times 100\;\\ & & \mathrm{FS}\left(\%\right)=\left(\text{residual volume after}\;3\;\min\right)\\ & & /\left(\mathrm{total}\;\mathrm{foam}\;\mathrm{volume}\right)\times 100 \end{array}$$

### 2.8 Emulsifying properties

Emulsifying properties were analyzed according to the methods described by Pearce and Kinsella (1978). Pure sunflower oil (15 mL) was mixed with 45 mL of a protein suspension 0.1% (w/v). The mix was then homogenized with a mixer at maximum speed for one minute. Fifty microliters of emulsion (the bottom of the homogenized emulsion) were withdrawn and diluted with 10 mL of 0.1% SDS solution. The absorbance was read at 500 nm. The emulsifying activity index (EAI) and the emulsion stability index (ESI) were calculated using the absorbance's at 0 and 10 min after the formation of the emulsion.  The emulsifying properties were expressed as EAI and ESI following the equations:

$$\begin{array}{ccc} \mathrm{EAI}\left(m^{2}/g\right) & = & \left(2T\times A_{0}\times F\right)\;/\;\left(C\times \Phi \times 10000\right)\;\mathrm{ESI}\;\left(\min\right)\\ & & =\;A_{0}\times \Delta t\;/\;\Delta A \end{array}$$

Where *T* = 2.303 A0/l; A0 = absorbance measured immediately after emulsion formation; F‐dilution factor = 200, l = path length of the cuvette, C = weight of protein/unit volume (g/mL) of aqueous phase before emulsion formation; Φ = oil volume fraction of the emulsion; Δ*t* = 10 min; Δ*A* = *A*_0_−*A*_10_Where A_10_ = absorbance measured after 10 minutes

### 2.9 WHC

Water holding capacity (WHC)Amino acids content of proteins (g/100 g proteins) from malt rootlets (MR), malt rootlets pellet (MRP), soy, pea and rice was determined according to AACC (2000c) method 56–30 with slight modification following the methods reported by Boye et al. (2010). Enough water was added to over saturate the sample but not too much to cause a liquid dispersion to form. The hydrated samples were centrifuged at low speed (2000 × g) and the supernatant was removed. WHC was expressed as the amount of water absorbed by 1 g of concentrate.

### 2.10 Fat Absorption Capacity

Fat absorption capacities were determined using the procedure by Lin et al. (1974) with slight modifications. Samples (0.5 g) were mixed with 3 mL of corn oil in a pre‐weighed 15 mL graduated centrifuged tube for 1 min. After centrifugation at 4000 g for 30 min, the supernatant was discarded, and the tubes were re‐weighed. The % FAC was calculated as follows:

$$\begin{array}{ccc} \mathrm{FAC}\left(\%\right) & = & 100\times \left(\mathrm{weight}\;\mathrm{of}\;\mathrm{fat}\;\mathrm{absorbed}\;\mathrm{by}\;\mathrm{sample}\right.\\ & & \left./\;\mathrm{weight}\;\mathrm{of}\;\mathrm{sample}\right) \end{array}$$

### 2.11 Statistical Analysis

Triplicate data were statistically evaluated by one‐way analysis of variance (ANOVA) using the PRISM software, version 10.0 (Graph Pad Software, Inc., San Diego, CA, USA). Significant differences between means were determined by the Tukey's multiple comparison test procedure at the 5% significance level (*p* < 0.05).

## 3 Results and Discussion

### 3.1 Protein Characterization

The extraction of malt rootlet proteins in an alkaline environment evidenced that the purity of MR and MRP was found to be highest at pH 11, with values of 70.6% (±2.74) and 62.8% (±1.45), respectively, coupled with a yield, on average, equal to 10%. These data differed from those reported by Cheng et al. (2016), conducting the extraction of proteins from barley MR at pH = 9, obtaining a final yield of of 29.1%, reaching a lower purity equal to 33.7 %.

**FIGURE 1:**
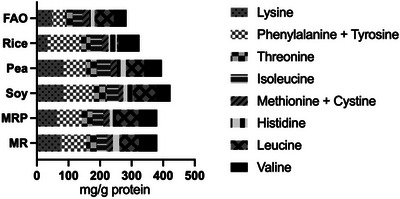
Essential amino acids content (mg/g protein) in protein samples from malt rootlets (MR), malt rootlets pellet (MRP), soy, pea and rice compared with the FAO requirement pattern (FAO, 2011).

The assessment of protein quality focuses on evaluating the ability of dietary protein sources and overall diets to fulfill the body's requirements for protein and essential amino‐nitrogen. The amounts of essential amino acids (∼38 g/100 g protein) for MR and MRP met the amino acid scoring pattern established by the FAO (Figure 1; Table 1), except for the sulphur‐containing amino acids (methionine + cysteine) that were found in lower quantities than the reference value of 23 mg/g (1.93 g/100 g protein for MR and 1.87 g/100 g protein in MRP), similarly to soy and pea proteins, thus resulting to be limitant (FAO 2011). The results showed MR and MRP were a good source of lysine (7.61 and 6.36 g/100 g protein), with higher content than the FAO reference value (48 mg/g protein), outdoing the amount reported in rice proteins (3.39 g/100 g protein). Thus, using barley malt rootlet proteins in cereal‐based foods may represent a valuable plant‐based alternative for significant accomplishment in terms of quali‐quantitative amino acids profiles of products (Messia et al. 2021, 2023; Waters et al. 2013, Salama et al. 1997).

**TABLE 1:** Amino acids content of proteins (g/100 g proteins) from malt rootlets (MR), malt rootlets pellet (MRP), soy, pea and rice.

−	MR	MRP	Soy	Pea	Rice
Essential amino acids	−	−	−	−	−
Lysine	7.61 ± 0.51a	6.36 ± 0.56a	8.42 ± 2.89a	8.44 ± 2.39a	3.39 ± 0.29b
Phenylalanine	5.32 ± 0.22a, b	4.64 ± 0.21b	4.86 ± 0.54a, b	4.26 ± 0.50c, b	3.86 ± 0.17c
Tyrosine	2.71 ± 1.39b	3.20 ± 0.73b	4.77 ± 0.54a	2.98 ± 0.43b	3.49 ± 0.56b
Threonine	3.35 ± 0.16c	3.40 ± 0.28c	4.07 ± 0.22a	3.40 ± 0.08b	2.95 ± 0.19c
Isoleucine	3.13 ± 0.24c	3.50 ± 0.15c	3.68 ± 0.06a, b	4.30 ± 0.08a	3.59 ± 0.04b
Methionine	1.03 ± 0.05b	0.89 ± 0.15b	0.47 ± 0.02c	0.82 ± 0.05b	1.15 ± 0.04a
Cystine	0.9 ± 0.17a	0.98 ± 0.09a	1.04 ± 0.20a	1.19 ± 0.69a	0.91 ± 0.01a
Histidine	2.10 ± 0.24b	2.03 ± 0.44b	3.12 ± 0.15a	2.84 ± 0.27a	2.77 ± 0.38a
Leucine	6.20 ± 0.27c	7.29 ± 0.04b, c	7.21 ± 0.99a	5.81 ± 0.31a, c	7.80 ± 0.39a, b
Valine	5.75 ± 0.28a	5.82 ± 0.05a	4.66 ± 0.27b	5.59 ± 0.27a, b	6.51 ± 0.19c
∑EAA	38.01	38.13	42.30	39.63	36.42
−	−	−	−	−	−
Non essential amino acids	−	−	−	−	−
Serine + Proline	9.65 ± 1.11c	11.07 ± 0.39bc	12.05 ± 0.77a	9.60 ± 0.12b	10.03 ± 0.56b, c
Glycine	4.75 ± 0.19b, a	4.79 ± 0.32b	4.28 ± 0.33b	4.78 ± 0.43b	5.66 ± 0.41a
Alanine	5.37 ± 0.67c	5.36 ± 0.53c	5.60 ± 0.01b	4.80 ± 0.22b,c	7.62 ± 0.08a
Glutamic Acid	14.05 ± 0.89b	21.39 ± 2.23a	9.83 ± 1.58c	21.49 ± 1.10a	14.8 ± 0.70b
Aspartic Acid	17.70 ± 2.08b	18.06 ± 0.49b	20.40 ± 1.78a	7.99 ± 0.40c	12.69 ± 0.69d
∑NEAA	51.51	60.67	52.16	48.64	50.47

*Note*: ΣEAA sum of all essential amino acids, ΣNEAA sum of all non‐essential amino acids. Values represent mean ± SD. Means with the same small letters, within the same row, are not significantly different at *p* < 0.05.

**FIGURE 2:**
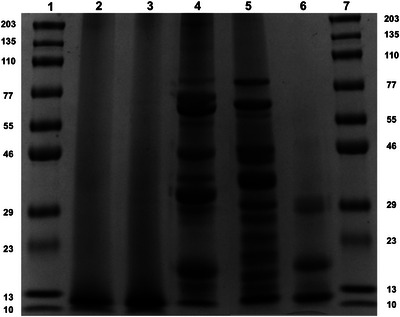
SDS‐PAGE of proteins from, malt rootlets (MR) (2), malt rootlets pellet (MRP) (3), soy (4), pea (5), and rice (6); std (1,7).

Regarding protein characterization through SDS‐PAGE (Figure 2), MR and MRP showed only one band equivalent to 11.2 KDa and 10.9 KDa, respectively, denoting that the protein concentrates mainly consist of low molecular weight proteins and peptides. On the other hand, in commercial soy and pea samples, most of the bands fall within a molecular weight range of 70 to 20 kDa. In peas, the rather intense low molecular weight band at 11 kDa, corresponded to that observed in the MR and MRP samples. Rice, on the other hand, shows only three bands below 30 kDa, due to the low solubility of rice proteins, even though all the samples have been pre‐solubilized before the run using a Tris‐HCl buffer (pH 9.5) with 1% SDS.

**FIGURE 3:**
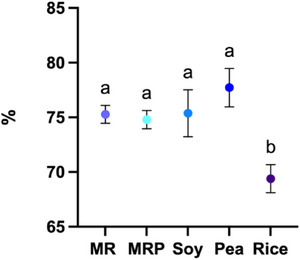
In vitro protein digestibility (IVPD) of protein samples from malt rootlets (MR), malt rootlets pellet (MRP), soy, pea and rice. Error bars refer to SD, means (average of three replicate data) with the same small letters are not significantly different at *p* < 0.05.

It is widely acknowledged that the ileal digestibility of plant proteins is typically lower compared to animal proteins. However, this can be enhanced by removing plant cell wall components or by isoelectric protein precipitation (Tomè, 2013; Barbana and Boye 2013). The IVPD data (Figure 3) showed no significant differences among MR, MRP, soy, and pea isolates, with respective values of 75.2%, 74.8%, 75.4%, and 77.7% in line with other studies of IVPD on legumes isolates (Aryee and Boye 2016; In et al. 2007; Nguyen et al. 2015). Only rice was found to have significantly lower digestibility, at 69.4%.

### 3.2 Functional Properties

#### 3.2.1 Protein Solubility

**FIGURE 4:**
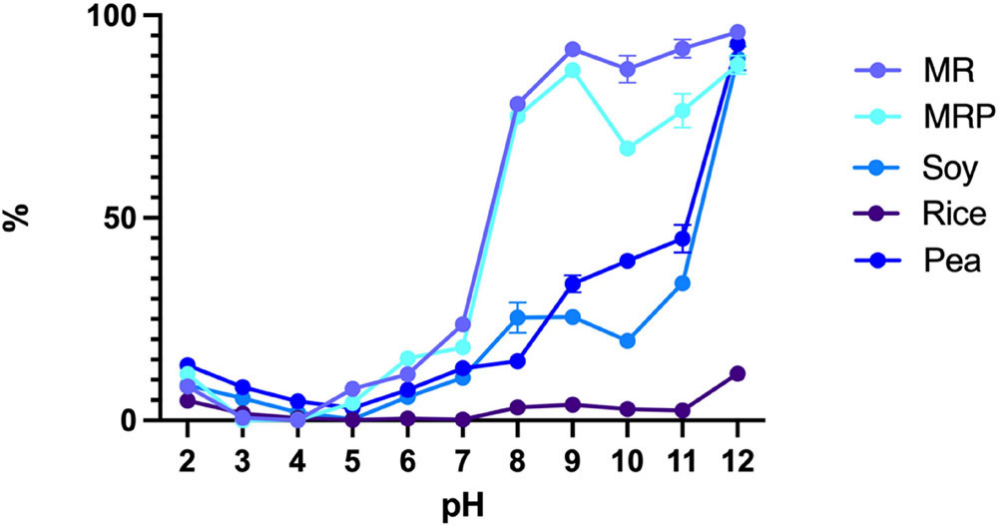
Relative solubility (%) at pH 2‐12 of proteins sample of malt rootlets (MR), malt rootlets pellet (MRP), soy, pea and rice. Error bars refer to SD; means are average of three replicate data.

Solubility is a key physicochemical property that influences a range of protein functionalities. In food production, solubility determines the types of products that can incorporate protein powders (e.g., solid or liquid), the phases that can be stabilized (oil or air), the required processing methods (such as thermal treatment, size reduction, or mixing) and their duration (Williams and Phillips 2021). The solubility characteristics of protein ingredients are influenced by extraction parameters such as solvent type, pH, ionic strength, mechanical forces, and temperature (McClements and Grossmann 2021). Additionally, other components in protein powders ‐like starch, dietary fibers, minerals, and lipids—can also affect solubility. In this study, relative solubility of proteins extracted from Barley MR was determined at various pH values between 2 and 12 (Figure 4). The same solubility trend was recorded for MRP and MR, similar to soy and pea protein. (Barac et al. 2015; Zhao et al. 2020; Ma et al. 2022). The isoelectric point, where the solubility is close to zero, of MR and MRP was found at pH3–4, while for soy, pea in the pH range 4–5. Then, alkaline pH progressively enhanced solubility, with a sharper increase recorded in rootlet samples compared to soy and pea protein, reaching 98.1% and 84.5% at pH 12 in MR and MRP, respectively. These data confirm the results by Salama et al. (1997), describing for malt rootlets a solubility of about 20% at pH 6–7, while rising pH conditions (8–12) lead to almost complete solubilization. On the other hand, MR and MRP proteins behaved differently from rice, that showed a solubility trend being close to zero at all points analyzed, despite reaching a maximum of 11% at pH 12. This coincided with results present in the literature (Zhao et al. 2020) and could be due to the fact that rice proteins are made for the 80% of glutelins, which are water insoluble because of their extensive aggregation, disulfide bond cross‐linking and glycosylation (Day 2013).

#### 3.2.2 Foaming Properties

**FIGURE 5:**
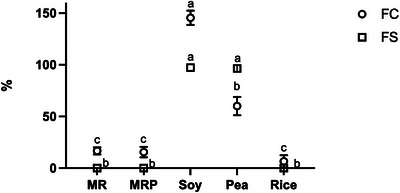
Foaming capacity (FC, as percentage of foam volume immediately after whipping) and Foaming stability (FS, as percentage of foam volume after three minutes from whipping) of malt rootlets (MR), malt rootlets pellet (MRP), soy, pea and rice proteins. Since foaming capacity of malt rootlets (MR), malt rootlets pellet (MRP) and rice was very low, the relative protein stability was not detectable. Error bars refer to SD, means (average of three replicate data) with the same small letters are not significantly different at *p* < 0.05.

Foaming ability is the property related to foam‐forming capacity. Foam is composed of gas bubbles dispersed in solid or liquid compounds (Heredia‐Leza et al. 2022) and high water solubility of proteins is one of the main drivers to achieve good foaming ability. Indeed, this causes the reduction of interfacial tension and forms strong, elastic films around the dispersed air bubbles (Murray 2007). Despite the solubility behavior of MR and MRP being similar to that of soy and peas, their FC was significantly lower, around 15–16 % (Figure 5). FC of soy protein was the highest (145.5%), followed by peas, which was almost half (60%), possibly due to the different structure and composition of the proteins and their related surface (Amagliani et al. 2021). Regarding FS, the value for MR and MRP was near to zero, also for the rice, while soy and pea showed good stability properties at approximately 96%. According to Ma et al. (2022), proteins with a relatively high content (above 90%) exhibited greater FS, indicating that increased protein concentration promotes this capacity. This could explain the inability of MR and MRP samples to maintain the foam once it is formed, due to the lower protein content in comparison to pea and soybean isolates.

#### 3.2.3 Emulsifying Properties

**FIGURE 6:**
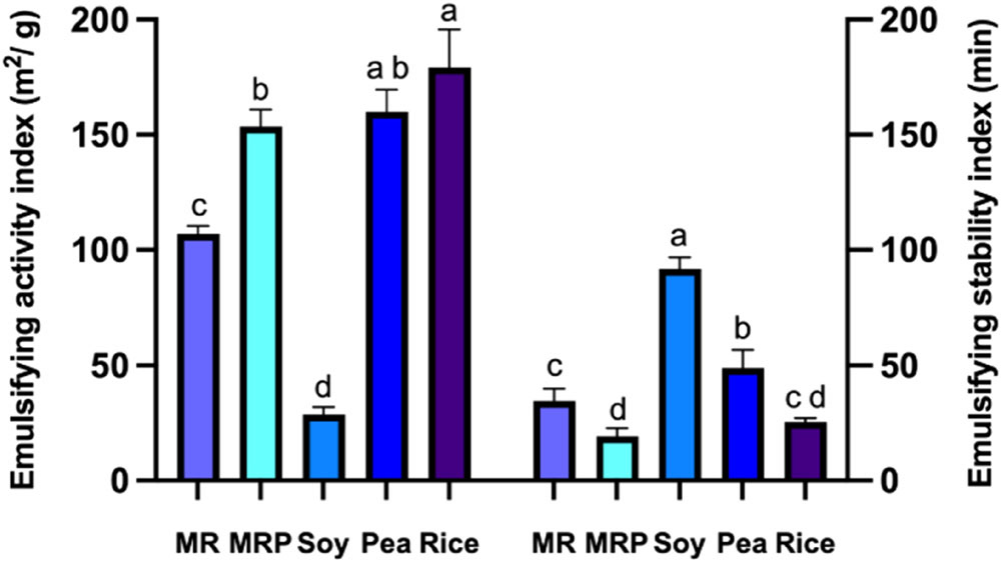
Emulsifying activity index (EAI) and Emulsifying stability index (ESI) of malt rootlets (MR), malt rootlets pellet (MRP), soy, pea and rice proteins. Error bars refer to SD, means (average of three replicate data) with the same small letters are not significantly different at *p* < 0.05.

The emulsifying properties of proteins are typically defined by their capacity to create and maintain stable emulsions, an attribute that is influenced by various factors, such as the size, shape, flexibility, charge, hydrophobicity, and aggregation state of protein molecules. Significant variability in emulsifying activities (EA) is observed across different studies, even when the same EA method is employed. However, the distinction between plant‐based proteins is not substantial (Ma et al. 2022). Emulsifying properties of the protein samples are reported in Figure 6. MR and MRP showed an emulsifying activity index (EAI) of 106.8 m^2^/g and 153.5 m^2^/g respectively, with the latter being equivalent to that of pea (160 m^2^/g) demonstrating good emulsifying activity (Figure 6). On the other hand, the ability of rootlets protein samples to maintain stable emulsions was higher than soy proteins (EAI = 28.68 m^2^/g), this could be explained by the fact that emulsifying properties were evaluated in water, which may lead to a change of pH depending on the type of dissolved protein. In fact, in the range of pH 5–7 the solubility of soy protein isolated resulted to be lower than MR, MRP, and pea. For once concerning the emulsifying stability index (ESI), neither the MR (34.7 min) nor MPR (19.3 min) showed optimal properties comparable to those of pea (48.9 min) and soy (91.7 min) protein. .Due to the low solubility at all pH values, the emulsifying properties of rice proteins were expected to be minimal according to the literature (Zhao et al. 2020), but the EAI of rice resulted in being the highest value, equivalent to those of the pea.

#### 3.2.4 Water Holding Capacity and Fat Absorption Capacity

The water holding and fat absorption capacities (WHC and FAC) of proteins measure how much water or oil they can hold per unit mass, respectively (Ma et al. 2022).

**FIGURE 7:**
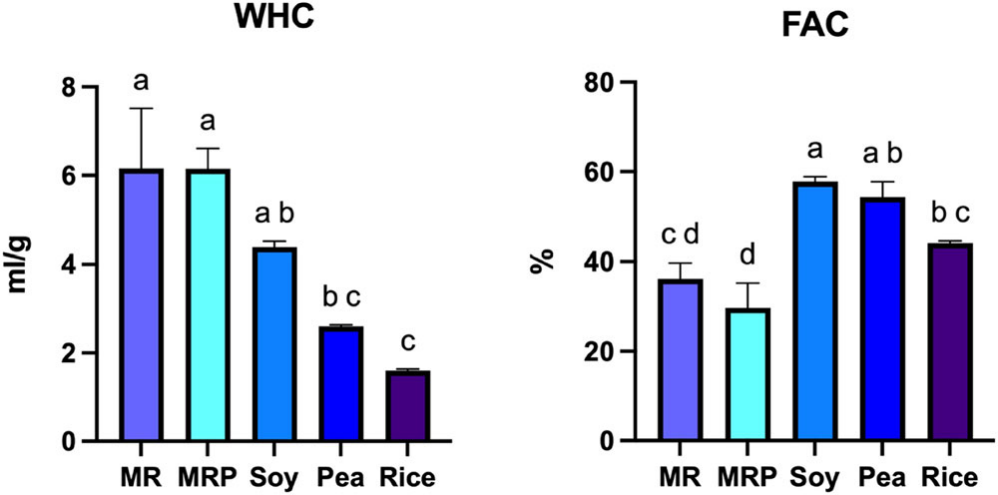
Water holding capacity (WHC) and Fat absorption capacity (FAC, calculated as percentage of weight gained) of protein from malt rootlets (MR), malt rootlets pellet (MRP), soy, pea and rice. Error bars refer to SD, means (average of three replicate data) with the same small letters are not significantly different at *p* < 0.05.

The WHC of MR and MRP resulted to be highest, equal to 6.15 mL/g, which was more than two times the capacity of pea protein (2.60 mg/mL), and five times that of rice protein (1.60 mg/mL) still outdoing also soy protein (4.38 mg/mL) (Boye et al. 2010; L'Hocine et al. 2006; Tang et al. 2021) (Figure 7). The data of FAC instead resulted to be opposite to WHC. Indeed, MR and MRP resulted to be the lowest (39.19% and 29.73%), followed by rice (43.24%), while soy and pea protein had the highest values (58.93% and 50.86%, respectively).

## 4 Conclusions

Protein concentrate extracted from barley malt rootles (MR) and malt residual pellet (MRP, including rootlets, cereal fines, husks, and small broken malted cereal grains), with an alkali method, presented a favorable amino acid profile with appreciable levels of lysine (7.61 and 6.36% total protein for MR and MRP, respectively), making them nutritionally promising. Digestibility data indicate that MR and MRP were on par with soy and pea proteins (∼75%), underscoring their potential for incorporation into plant‐based dietary products. Their protein solubility aligns closely with that of soy and pea proteins but shows lower foaming capacity and emulsifying stability, likely due to different protein content and structural characteristics. Despite this, MRP exhibited good emulsifying activity comparable to pea protein. Furthermore, the water‐holding capacity of MR and MRP outperforms other isolates, suggesting a potential application in semi‐solid formulations.

Overall, no significant differences were recorded among nutritional and functional properties of the two types of by‐products: rootlets obtained after the deculming phase, and the rootlets pellet. Thus, MRP, representing the true industrial by‐product of the malting process, may offer a sustainable and valuable protein source. Their favorable amino acid composition, digestibility, and functional properties suggest potential applications in the food industry. Specifically, their high water‐holding capacity makes them suitable for semi‐solid formulations, while their emulsifying properties could enhance plant‐based dairy or meat alternatives. Altogether, results suggest that malting by‐products could be effectively re‐used to produce protein‐rich ingredients to improve the nutritional and functional quality of various foods, also from the perspective of industrial scale‐up for protein recovery.

## Author Contributions

**Niccolò Pilla**: conceptualization, investigation, methodology, formal analysis, data curation, writing – original draft. **Elisa De Arcangelis**: conceptualization, writing – original draft, methodology, formal analysis. **Gianfranco Pannella**: formal analysis, writing – review and editing. **Emanuele Marconi**: supervision, writing – review and editing, conceptualization.

## Conflicts of Interest

The authors declare no conflicts of interest.

## Data Availability

Data available on request.
